# Reproductive Toxicities Caused by Swainsonine from Locoweed in Mice

**DOI:** 10.1155/2016/6824374

**Published:** 2016-11-24

**Authors:** Chenchen Wu, Ke Feng, Dezhang Lu, Dujian Yan, Tiesuo Han, Baoyu Zhao

**Affiliations:** ^1^College of Animal Veterinary Medicine, Northwest A&F University, Yangling, Shaanxi 712100, China; ^2^Lhasa City Animal Disease Prevention Control Center, Lhasa 851000, China; ^3^AKS Vocational and Technical College, Aksu, Xinjiang 843000, China; ^4^Animal Health Center, Lanzhou Chia Tai Food Co., Ltd., Charoen Pokphand Group, Lanzhou, Gansu 730200, China

## Abstract

Swainsonine is the primary toxin in locoweeds. It causes intention tremors, reproductive dysfunction, emaciation, and death. The objective of the present study was to evaluate the potential reproductive and developmental toxicities caused by swainsonine in mice. The treatment groups consisting of three generations of mice were given a range of concentrations of swainsonine by intraperitoneal injection (2.50 mg/kg body weight (BW), 1.20 mg/kg BW, 0.60 mg/kg BW, and 0 mg/kg BW). The 0 mg/kg BW group exhibited significantly fewer estrous cycles and an increased number of estrous ones compared to the 2.50 mg/kg BW, 1.20 mg/kg BW, and 0.60 mg/kg BW groups (*P* < 0.05). All three generations of mice treated with swainsonine had significantly higher spleen, liver, and kidney indices and significantly lower body weights compared to the 0 mg/kg BW group (*P* < 0.05). For the first and second generations of treatment group, the copulation indices and the numbers of live pups on postnatal days (PND) 0, 4, and 15 were significantly decreased compared to those of the 0 mg/kg BW group (*P* < 0.05). The fertility and gestation indices of the treatment group of the first generation were significantly increased compared to the 2.50 mg/kg BW, 1.20 mg/kg BW, and 0.60 mg/kg BW groups of the second generation (*P* < 0.05). Cumulatively, these results indicate that swainsonine may cause reproductive and developmental toxicities in mice in both parents and offspring.

## 1. Introduction

Locoweeds (*Astragalus* and* Oxytropis* spp.), a taxa of the Legume family, are toxic plants in the western United States that frequently poison livestock [1]. Consumption of locoweeds by grazing animals can result in locoism, which is characterized by emaciation, staggering gait, lack of muscular coordination, reproductive disturbances, immune system impairment, and death [2, 3]. The economic cost owing to locoism reaches several million dollars per year due to death, abortion, reproductive problems, and lack of weight gain [4–6]. Swainsonine, an indolizidine alkaloid, is the primary toxin in locoweeds [3]. Swainsonine is an effective inhibitor of both lysosomal *α*-mannosidase and Golgi *α*-mannosidase II [7]. Swainsonine toxicity caused by consumption of locoweed causes intention tremors, generalized depression, nervousness, proprioceptive deficits, aberrant behavior, reproductive dysfunction, emaciation, and death [8]. Additionally, locoweeds cause embryonic and fetal lethality, abortions, generalized reproductive dysfunction, and occasional birth defects. Swainsonine from locoweed causes acute intoxication in horses, goats, and sheep, in addition to chronic poisoning in rabbits, rats, and mice. Its toxicity often occurs during pregnancy in livestock and may have different effects on embryonic development depending on the conceptus phase and maternal conditions during acute intoxication. Therefore, we observed swainsonine effects on mouse reproductive performance during chronic poisoning. The objective of the present study was to evaluate the effect of three different doses of swainsonine in female mice during estrus, gestation, childbirth, and lactation. We also treated the first (F0), second (F1), and third (F2) generations of mice with three different doses of swainsonine during growth and development. We report the results of the reproductive and developmental toxicities of swainsonine.

## 2. Material and Methods

### 2.1. Animals and Housing Conditions

Female Wistar mice (6 weeks old) were supplied by the Animal Center of the Fourth Military Medical University. During the experiment, the animals were housed individually in polypropylene cages with laboratory-grade pine shavings as bedding. The mice were maintained under controlled temperatures (±23°C). Lighting conditions consisted of a 12 : 12 hour light : dark cycle in a controlled environment with >8 air exchanges/hour and temperature and relative humidity in the ranges of 19–25°C and 40–70%, respectively. The experimental procedures were in accordance with the Ethical Principles in Animal Research adopted by the China College of Animal Experimentation and were approved by the College of Veterinary Medicine Northwest A&F University.

### 2.2. Extraction of Swainsonine from Locoweed

#### 2.2.1. Plant Materials

The aerial portion of* Oxytropis kansuensis* was collected from a grassland in Tianzhu City of Gansu province in July 2011. The plants were then taxonomically identified by Zhao Bao-Yu, College of Veterinary Medicine, Northwest A&F University, China. The plants were subsequently dried in the shade, finely ground, and comminuted.

#### 2.2.2. Isolation of Alkaloids from* Oxytropis kansuensis*

The plant sample (1.5 kg) was thermally refluxed in 10 L of distilled water into an ultrasonic cleaner for approximately 12 hours. The sample was subjected to heat treatment for approximately 1 hour at 50°C in an ultrasonic cleaner. A total of 18 L of the water extract was collected and reduced over heat to 2 L. The solution was centrifuged at 1500 r/min for 10 min and the solid impurities were removed. The sample was then concentrated to 1 L at 95°C, followed by recovery of the EtOH under reduced pressure to obtain a crude extract for isolation of the alkaloids. The extract was dissolved in 1 N HCl, and the filtrate was extracted with chloroform and alkalized. The aqueous fraction was successively extracted with chloroform, ethyl acetate, and n-butanol. The raw alkaloids were collected with n-butanol, dissolved in methyl alcohol, and filtered. The residuals were removed. The solvents were recovered using methanol and pressure, and the extractions with alkaline chloroform were repeated. We collected crude swainsonine with chloroform. We used silica gel column chromatography for purification. For the dry sample, we used a chloroform-methyl alcohol-water gradient elution and thin-layer chromatography (TLC) detection. For the collection of swainsonine, the extract was decompression-drained. Finally, a light yellow powder of swainsonine was collected, and pure swainsonine was obtained using a fractional distillation method [9].

#### 2.2.3. Analysis of Swainsonine

TLC detection was performed on plates precoated with silica gel G using developing solvents (chloroform : methanol : ammonia : water [70 : 26 : 2 : 2, v/v], chloroform : methanol : ammonia : water [70 : 26 : 10 : 10, v/v], and methanol : ethyl acetate : ammonia [4 : 1 : 1, v/v]) and either a modified potassium heptaiodobismuthate reagent or H_2_O_2_/10% acetic anhydride in EtOH/Ehrlich's reagent as the chromogenic agent.

The extracts were dissolved in methanol and spotted onto the GF254 silica gel G precoated plates. The plates were developed with an ascendant run after saturation with the mobile phase in an s glass chamber for 5–10 min. The plates were dried when the mobile phase was 10 mm from the front edge of the plates. The plates were stained successively with a spray of H_2_O_2_ (heated for 10 min in an oven at 115°C), a spray of 10% acetic anhydride in dehydrated alcohol (heated at the same temperature until the smell of acetic anhydride disappeared), and finally a spray of Ehrlich's reagent (heated for 15 min at 120°C). The color of the spots in each plate was recorded, and the Retardation factor (*R*_*f*_) was determined [9].

### 2.3. Study Design

To assess the reproductive and developmental toxicity of swainsonine, three generations of mice were used. The study design was as follows.

#### 2.3.1. Experiment 1: Observation of the Estrous Cycle

Female mice (*N* = 40, 6 weeks old, 10 animals per group, consisting of 2.50 mg/kg BW, 1.20 mg/kg BW, 0.60 mg/kg BW, and 0 mg/kg BW mice) were treated 14 days before mating and throughout the mating period. After 14 days of treatment, 40 female mice were observed for the estrous cycle using the vaginal smear method. The vaginal lavage sample from each female was evaluated daily for the estrous cycle during the next 15 days of treatment. We collected the reproductive organs and recorded the estrous parameters after 15 days of treatment. Estrous cycles of 4-5 days were considered normal, and any other cycle lengths were considered irregular. In particular, cycles with more than 7 days of diestrus were considered continuous diestrus [10].

#### 2.3.2. Experiment 2: The Observation of Reproductive Performance Test

Female mice (*N* = 40, 6 weeks old) were divided into four groups (10 animals per group: F0-I: 2.50 mg/kg BW; F0-II: 1.20 mg/kg BW; F0-III: 0.60 mg/kg BW; and F0-IV: 0 mg/kg BW). The F0-IV group was considered the control group, and the other three groups were the treatment groups.

Mice were given swainsonine by intraperitoneal injection 14 days before the mating period. After this premating period, 40 female mice (10 per group) were transferred to the home cage of male mice in the same group and cohabited on a 1 : 1 basis until a mating period of 2 weeks elapsed. During the mating period, vaginal plugs were examined daily for the presence of sperm, and a vaginal plug was considered evidence of successful mating. The day of successful mating was designated as day 0 of pregnancy. We recorded pregnancy rates of the female mice. A confirmed pregnant mouse continued to receive swainsonine throughout the parturition and lactation periods. When the newborn mice began weaning, the female mice (F0) were sacrificed, and the liver, kidney, heart, spleen, lungs, uterus, and ovaries were collected.

Newborn female mice (F1) were treated with swainsonine after delactation (40 F1 mice total, 10 per group: F1-I: 2.5 mg/kg BW; F1-II: 1.20 mg/kg BW; F1-III: 0.60 mg/kg BW; and F1-IV: 0 mg/kg BW). They were selected from the four groups of the F0 generation. When the F1 mice were 8 weeks old, they were mated with healthy male mice and cohabited on a 1 : 1 basis until a mating period of 2 weeks elapsed. All F1 mice were treated with swainsonine before mating and throughout the mating, gestation, and lactation periods. When newborn mice began weaning, the F1 female mice were sacrificed, and the liver, kidney, heart, spleen, lungs, uterus, and ovaries were collected.

Newborn female mice (F2) were selected from each of the four groups of the F1 generation. The F2 mice were not treated with swainsonine until delactation. They were then sacrificed, and the liver, kidney, heart, spleen, lungs, uterus, and ovaries were collected.

All experimental animals received intraperitoneal injections of swainsonine once every 3 days under aseptic condition. For each mouse that was sacrificed, the liver, kidney, heart, spleen, lungs, uterus, and ovaries were trimmed of extraneous fat and weighed immediately. The organ weight and terminal body weights were recorded.

### 2.4. Data Analysis

Statistical Product and Service Solutions (SPSS) software v11.3 was used to determine whether there were any statistically significant differences between the test groups and the control group. A one-way ANOVA was used to evaluate the data, and Dunnett's multiple comparison test was then applied. Values of *P* < 0.05 were considered significant. The data are presented as the group mean values ± standard deviation (SD).

## 3. Results

### 3.1. TLC Detection

**Figure 1:**
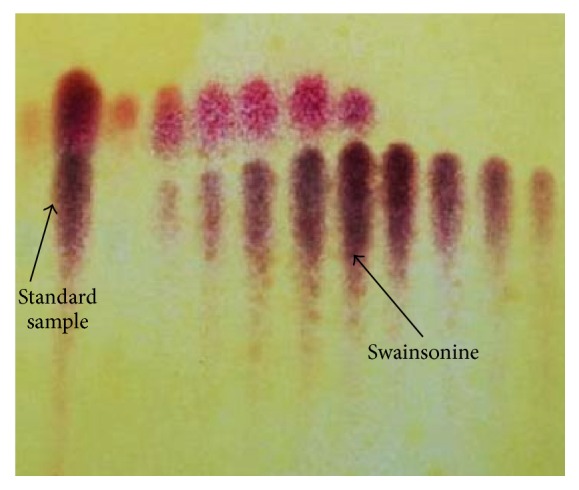
Thin-layer chromatography of swainsonine. The standard swainsonine sample (left arrow). Swainsonine is represented by the purple spots (right arrow).

All extracts were collected using column chromatography, which was placed on the thin-layer plate using the capillary sample.  Figure 1 shows a developed TLC plate. The purple spots are swainsonine and the rose red spots are the swainsonine analogs as determined by comparison with the swainsonine standard (Retardation factor [*R*_*f*_] = 0.50).

### 3.2. Experiment 1: Body and Reproductive Organ Weights during the Observation of the Estrous Cycle

The body and reproductive organ weights increased for each group during the estrus cycle, as reported in Table 1. After 14 days of swainsonine treatment, we began to observe the estrus cycle for the four groups of mice over the course of 15 days. The mice were given swainsonine once every 3 days.

The body weights of the 2.50 mg/kg BW, 1.20 mg/kg BW, and 0.60 mg/kg BW groups were significantly lower than those of the 0 mg/kg BW group after 7 and 15 days of swainsonine treatment (*P* < 0.05). Furthermore, the body weights of the 2.50 mg/kg BW group were significantly lower than those for the 1.20 mg/kg BW and 0.60 mg/kg BW groups at 7 and 15 days of treatment (*P* < 0.05).

The ovarian index of the 2.50 mg/kg BW and 1.20 mg/kg BW groups of mice was significantly decreased compared to that of the 0.60 mg/kg BW and 0 mg/kg BW groups at 7 and 15 days of treatment. The ovarian index of the 0.60 mg/kg BW group was lower than that for the 0 mg/kg BW group (*P* < 0.05). The ovarian index decreased from 0 mg/kg BW group to 2.50 mg/kg BW group.

The uterine index of the 2.50 mg/kg BW, 1.20 mg/kg BW, and 0.60 mg/kg BW groups of mice was significantly decreased compared to that of the 0 mg/kg BW group at 7 days of treatment (*P* < 0.05). The uterine index of the 2.50 mg/kg BW, 1.20 mg/kg BW, and 0.60 mg/kg BW groups was not significantly different from each other at 7 days of treatment (*P* > 0.05). However, the 2.50 mg/kg BW and 1.20 mg/kg BW mice displayed a uterine index lower than that of the 0.60 mg/kg BW and 0 mg/kg BW groups at 15 days of treatment (*P* < 0.05). The uterine index for the 0.60 mg/kg BW mice was also significantly lower than that of the 0 mg/kg BW group at 15 days of treatment (*P* < 0.05).

With respect to the length of the estrous cycle (in days), the 2.50 mg/kg BW and 1.20 mg/kg BW groups displayed longer cycles than those of the 0.60 mg/kg BW and 0 mg/kg BW groups at 7 days of treatment (*P* < 0.05). The 0.60 mg/kg BW mice had significantly longer cycles compared to those of the 0 mg/kg BW group (*P* < 0.05). The estrous cycle length increased for the 2.50 mg/kg BW group compared to that of the 0 mg/kg BW group at 7 days of treatment. The 2.50 mg/kg BW group had significantly longer cycles compared to those of the 1.20 mg/kg BW, 0.60 mg/kg BW, and 0 mg/kg BW groups at 15 days of treatment (*P* < 0.05). The 1.20 mg/kg BW and 0.60 mg/kg BW groups also had longer cycles than those of the 0 mg/kg BW group, and there was a significant difference between the 1.20 mg/kg BW and 0.60 mg/kg BW groups at 15 days of treatment (*P* < 0.05).

The number of mice experiencing estrous cycle was lower in the 2.50 mg/kg BW and 1.20 mg/kg BW groups than in the 0.60 mg/kg BW and 0 mg/kg BW groups at 7 days of treatment (*P* < 0.05). The number of mice undergoing estrous cycle in 0.60 mg/kg BW group was significantly fewer than that in the 0 mg/kg BW group (*P* < 0.05). This number also decreased from the 2.50 mg/kg BW group to 0 mg/kg BW group at 7 days of treatment. The 2.50 mg/kg BW group had significantly fewer mice experiencing estrous cycle than 1.20 mg/kg BW, 0.60 mg/kg BW, and 0 mg/kg BW groups at 15 days of treatment (*P* < 0.05). Finally, the 1.20 mg/kg BW and 0.60 mg/kg BW groups had fewer mice in estrous cycle than the 0 mg/kg BW group, and there was a significant difference between the 1.20 mg/kg BW and 0.60 mg/kg BW groups at 15 days of treatment (*P* < 0.05).

### 3.3. Experiment 2: The Reproductive Performance Test

#### 3.3.1. The Relative Organ Weights in Mice

The relative organ weights and body weights of the mice are shown in Table 2. The relative heart weight and relative lung weight of the four groups of mice from the F0, F1, and F2 generations were not significantly different among each group (*P* > 0.05).

For the relative liver weights of the F0, F1, and F2 generations, the experimental group had a significantly higher weight compared to that of the control group mice (*P* < 0.05). In the F0 and F1 generations, the liver weights of the mice in groups I, II, and III were significantly higher compared to those of the group IV mice (*P* < 0.05). There was no significant difference among mice of the groups I, II, and III (*P* > 0.05). For the F2 generation, the F2-III and F2-IV mice had significantly lower liver weights compared to those of groups F2-III and F2-IV (*P* < 0.05); however, there was no significant difference between the F2-I and F2-II groups and between the F2-III and F2-IV groups (*P* > 0.05). The relative liver weights of the F1 generation were higher than those of the F0 generation, except for the group IV mice.

For the spleen and relative kidney weights of the F0, F1, and F2 generations, the groups I, II, and III mice had significantly higher values compared to those of group IV (*P* < 0.05); however, there was no significant difference among groups I, II, and III (*P* > 0.05). The spleen and kidney indices of the F1 generation were higher than those of the F0 generation, except for the group IV mice.

For the relative ovary and uterus weights of the F0 and F1 generations, groups I, II, and III had significantly higher values compared to the group IV mice (*P* < 0.05). However, in the F2 generation, mice in groups I, II, and III had significantly lower values compared to those of the group IV mice (*P* < 0.05).

For body weights in the F0, F1, and F2 generations, groups I, II, and III were significantly lower compared to those of group IV (*P* < 0.05); however, there were no significant differences among groups I, II, and III (*P* > 0.05). The body weights of the F1 generation were lower than those of the F0 generation, except for the group IV mice.

#### 3.3.2. Reproductive and Developmental Index

The developmental findings are shown in Table 3. For the F0 and F1 generations, the number of live pups for groups I, II, and III decreased from PND 0 to PND 15. The number of live pups obtained from the F0-IV and F1-IV groups was not significantly different from PND 0 to PND 15. For the F0 and F1 generations, the number of live pups on PND 0 for groups I, II, and III was significantly lower compared to that of group IV (*P* < 0.05). The number of live pups on PND 4 for group IV was significantly higher compared to that of groups I, II, and III (*P* < 0.05). The number of live pups on PND 4 for group III was significantly higher compared to that of group I (*P* < 0.05). The numbers of live pups on PND 15 for groups III and IV were significantly higher compared to those of groups I and II (*P* < 0.05). The number of live pups on PND 15 for group IV was higher than that of group III (*P* < 0.05).

The body weights of the live mice in the F0 generation were higher than those for the F1 generation during the same period. For the F0 and F1 generations, the body weights of the live mice of groups I, II, and III were significantly lower compared to those of group IV on PND 0, 4, and 15 (*P* < 0.05). There was no significant difference observed among groups I, II, and III (*P* > 0.05).

The reproductive findings are shown in Table 4. For the F0 and F1 generations, the copulation index for group IV was significantly increased compared to that of groups I, II, and III (*P* < 0.05). The copulation index of group III was significantly increased compared to that of groups I and II (*P* < 0.05). For the F0 and F1 generations, there were no significant differences among groups I, II, III, and IV with respect to gestation length (*P* > 0.05). The fertility and gestation indices of groups F0-I, F0-II, and F0-III were significantly higher compared to those of groups F1-I, F1-II, and F1-III (*P* < 0.05). There was no significant difference between F0-IV and F1-IV (*P* > 0.05).

## 4. Discussion

In this study, we successfully isolated swainsonine from* Oxytropis kansuensis*. The cationic structure of swainsonine is similar to that of mannose, and its high affinity to mannosidase allows swainsonine to inhibit lysosomal *α*-mannosidase [11]. In animal cells, vacuole degeneration occurs upon swainsonine poisoning. Cellular vacuolation occurs in most tissues because of the incomplete degradation of the carbohydrate portion of glycoproteins and the accumulation of undressed oligosaccharides [12, 13]. The objective of this study was to determine the effects of swainsonine toxicity on reproduction and development in mice. Doses of 2.50 mg/kg BW, 1.20 mg/kg BW, and 0.60 mg/kg BW were given to mice, which were sufficiently high to induce general toxic effects in the parents and offspring. Furthermore, these dosages of swainsonine produced measurably toxic effects on estrus in these mice.

Previous studies suggest that long-term ingestion of plants containing swainsonine induces significant toxic effects on reproduction in livestock [14]. However, these studies did not demonstrate any effects of swainsonine on reproductive performance or the estrous cycle for three consecutive generations of mice. In experiment 1, after 14 days of swainsonine treatment, we observed estrus regulation for four groups of mice over 15 days with continued administration of swainsonine. Changes in the estrous cycles were observed for groups receiving 2.50 mg/kg BW, 1.20 mg/kg BW, 0.60 mg/kg BW, and 0 mg/kg BW treatments of swainsonine. A prolonged estrous cycle and decreased ovarian and uterine indices were observed after 15 days of treatment. The number of estrous mice decreased and there was a reduction in body weight after 15 days of treatment. The ingestion of plants containing swainsonine can decrease serum progesterone concentrations and subsequently disrupt ovarian function, accompanied by a delayed estrus cycle, increased estrous cycle length, delayed conception, and abortion [15]. We found that the number of estrous mice decreased and the estrous cycles were lengthened by the increasing accumulation of swainsonine in the body. Cumulatively, swainsonine affects the estrous cycle and the number of estrous mice, leading to dysfunctional estrous and no estrus performance.

In experiment 2, changes in organ weights were observed for the kidney, liver, and particularly the spleen in the F0, F1, and F2 generations. The kidneys displayed mean swainsonine concentrations of 154 ng/g when histologic lesions were first observed. Liver lesions were observed when the liver had a mean swainsonine concentration of 319 ng/g [16]. The weights of the livers in groups I, II, and III were higher than those of group IV; however, the weights of livers and kidneys in mice of the F1-I, F1-II, and F1-III generations were higher than those of the F0-I, F0-II, and F0-III generations. The same results were observed for the F0 and F1 generations, and the mice of the F2 generation displayed similar results. Swainsonine poisoning causes hepatomegaly and kidney enlargement. However, the changes in the spleen were more pronounced than the changes in the liver and kidneys. The weights of the spleens in the mice of the F1-I, F1-II, and F1-III generations were higher than those of the F0-I, F0-II, and F0-III generation. The mice of the F2 generation also displayed similar results. Monocytes and macrophage-like cells appear to be very sensitive to locoweed-induced changes [17]. Because the spleen contains lymphoreticular tissue, the immune cells in the spleen may also accumulate swainsonine. The macrophages and reticular cells in the spleen and other lymphoid tissues are some of the first cells to develop the characteristic vacuolation associated with locoweed poisoning [18, 19]. This pathological change is initially observed in these tissues because they accumulate swainsonine more readily than other tissues. The ability of these tissues to accumulate swainsonine primarily depends on differences in metabolism, and swainsonine exhibits varying affinities for different types of mannose [20–22]. Swainsonine is water-soluble and rapidly distributes into many parts of the body. In a previous study, swainsonine concentrations varied widely in different tissues and organs of sheep that ingested locoweed [23]. When livestock graze upon swainsonine-containing plants, the molecule accumulates at high concentrations in certain tissues and organs, which might impair their function and cause the typical symptoms associated with swainsonine poisoning.

Natural and experimental long-term ingestion of locoweed causes serious dysfunction in the reproduction of livestock (cattle, sheep, horses, and goats) in both males (mating behavior and libido) and females (failure to conceive, early embryo loss, or abortion) [24]. Ingestion of locoweed also decreases serum progesterone concentrations and subsequently disrupts ovarian function. This is accompanied by delayed estrus cycle, increased estrous cycle length, delayed conception, and abortion [25]. The weights of the ovaries and uterus for mice in groups I, II, and III were significantly higher compared to those of group IV for the F0 and F1 generations. However, the weights of the ovaries and uterus for mice in groups I, II, and III were significantly lower compared to those of group IV for the F2 generation. This result indicates that the ovaries and uterus of pregnant female mice are enlarged after swainsonine treatment, while those of nonpregnant female mice become smaller after swainsonine treatment. Therefore, swainsonine has a significant impact on the uterus and ovaries before and after pregnancy. We hypothesize that swainsonine causes shrinking of the ovaries and uterus, leading to disordered estrous cycle and decreased copulation and fertility rates before the pregnancy period. The higher concentrations of swainsonine significantly inhibited production of progesterone in a concentration- and time-dependent manner. The inhibition of progesterone production can be attributed to a reduction in luteal cell viability [26]. Flow cytometry and DNA fragmentation analyses indeed confirmed that higher concentrations of swainsonine inhibited luteal cell growth due to induction of cell cycle arrest and apoptosis [27]. Because swainsonine affects the estrus cycle, which leads to estrus dysfunction, the numbers of mating pairs displaying successful copulation for mice in groups I, II, and III were fewer compared to those of group IV for the F0 and F1 generations. The fertility index and gestation index for mice in groups I, II, and III of the F0 generation were significantly higher compared to those of the F1 generation. The number of pregnant female mice decreased with higher accumulations of swainsonine in the body. Furthermore, swainsonine caused several abnormalities, such as enlargement of the ovaries and uterus after the pregnancy period. We hypothesize that swainsonine will reduce the ability of female mice to conceive a second time, but there have been no reports of this thus far.

Feeding locoweed to pregnant ewes induces fetal cardiac dysfunction, delayed placentation, reduced placental and vascular development, hydrops amnii, abnormal cotyledonary development, interruption of fetal fluid balance, and abortion [28]. We showed that groups I, II, and III of the F0 and F1 generations of pregnant female mice birthed live pups, but the pups were weak or dead for groups I, II, and III of the F0 and F1 generations. The number of live pups on PND 15 for groups I, II, and III was significantly lower compared to those in group IV for the F0 and F1 generations. The body weights of the live pups on PND 15 of groups I, II, and III for F0 were higher than those of the F1 generation mice. Locoweed causes embryonic and fetal death, abortions, generalized reproductive dysfunction, and occasional birth defects [29]. In this study, the fetuses underwent autolysis, and the number of live pups on PND 0 for groups I, II, and III was lower than that of group IV for the F0 and F1 generations. Swainsonine exposure in utero also had severe effects on fetal goats, and the ultrasound observations were consistent with observed progesterone levels [30]. It is postulated that this cytoplasmic vacuolation interferes with the transport of nutrients across the placental barrier. Vacuolation itself may be associated with the death of the conceptus and subsequent abortion. A contributing factor may be that luteal cells of the ovary are also extremely vacuolated in ewes poisoned by locoweed and may not be producing sufficient progesterone to maintain pregnancy [31].

In conclusion, severe reproductive and developmental toxicities are associated with swainsonine poisoning. Specifically, swainsonine accumulation in the body prolonged the estrous cycle, decreased the number of live pups obtained on PND 0, 4, and 15, and reduced copulation and fertility indices in mice. Furthermore, the weights of the spleen significantly increased, and the body weights significantly decreased for mice in groups I, II, and III for the F0, F1, and F2 generations. Cumulatively, swainsonine toxicity significantly disrupts reproductive performance in the parent and offspring.

## Figures and Tables

**Table 1:** Body and reproductive organ weights of female mice given swainsonine during the estrous cycle.

		Body weight (g)	Ovarian index	Uterus index	Estrous cycle (day)	Number of estrous mice
Day 7	2.50 mg/kg BW	26.15 ± 1.14^a^	0.028 ± 0.002^a^	0.198 ± 0.004^a^	6.92 ± 0.23^a^	3.53 ± 0.21^a^
	1.20 mg/kg BW	28.04 ± 1.33^b^	0.029 ± 0.002^a^	0.208 ± 0.003^a^	6.54 ± 0.15^a^	4.01 ± 0.17^a^
	0.60 mg/kg BW	29.58 ± 1.55^c^	0.035 ± 0.002^b^	0.210 ± 0.003^a^	5.88 ± 0.14^b^	5.81 ± 0.21^b^
	0 mg/kg BW	31.41 ± 1.10^d^	0.048 ± 0.003^c^	0.300 ± 0.002^b^	4.20 ± 0.16^c^	7.54 ± 0.19^c^

Day 15	2.50 mg/kg BW	27.05 ± 1.66^a^	0.030 ± 0.002^a^	0.209 ± 0.003^a^	7.64 ± 0.25^a^	2.99 ± 0.24^a^
	1.20 mg/kg BW	28.89 ± 1.78^b^	0.031 ± 0.002^a^	0.216 ± 0.003^a^	6.91 ± 0.14^b^	3.98 ± 0.31^b^
	0.60 mg/kg BW	30.5 ± 1.25^c^	0.037 ± 0.003^b^	0.311 ± 0.004^b^	6.00 ± 0.15^c^	4.55 ± 0.25^c^
	0 mg/kg BW	32.19 ± 1.12^d^	0.051 ± 0.002^c^	0.364 ± 0.003^c^	4.45 ± 0.11^d^	7.66 ± 0.21^d^

The values are the mean ± SD.

Ovary viscera index = ovary weight/body weight × 100 (mg/g).

Uterus viscera index = uterus weight/body weight × 100 (mg/g).

^a,b,c,d^Significantly different from each dosage group of same generation (*P* < 0.05).

**Table 2:** Relative organ weights of mice given swainsonine by intraperitoneal injection.

	Heart index	Liver index	Spleen index	Lung index	Kidney index	Ovary index	Uterus index	Body weights (g)
F0-I	0.581 ± 0.05^a^	5.411 ± 0.88^a^	0.558 ± 0.06^a^	0.581 ± 0.04^a^	1.682 ± 0.21^a^	0.079 ± 0.002^a^	0.482 ± 0.02^a^	30.8 ± 1.22^a^
F0-II	0.588 ± 0.04^a^	5.401 ± 0.95^a^	0.535 ± 0.05^a^	0.587 ± 0.05^a^	1.674 ± 0.18^a^	0.076 ± 0.003^a^	0.479 ± 0.03^a^	31.7 ± 1.25^a^
F0-III	0.579 ± 0.06^a^	5.390 ± 0.85^a^	0.524 ± 0.05^a^	0.586 ± 0.06^a^	1.671 ± 0.23^a^	0.071 ± 0.002^a^	0.468 ± 0.02^a^	32.8 ± 1.31^a^
F0-IV	0.589 ± 0.05^a^	5.013 ± 0.92^b^	0.435 ± 0.06^b^	0.588 ± 0.05^a^	1.102 ± 0.19^b^	0.061 ± 0.003^b^	0.356 ± 0.03^b^	35.1 ± 1.55^b^
F1-I	0.598 ± 0.04^a^	5.448 ± 0.96^a^	0.561 ± 0.05^a^	0.583 ± 0.06^a^	1.692 ± 0.20^a^	0.081 ± 0.002^a^	0.498 ± 0.02^a^	29.3 ± 1.33^a^
F1-II	0.593 ± 0.03^a^	5.428 ± 0.89^a^	0.555 ± 0.06^a^	0.591 ± 0.05^a^	1.684 ± 0.22^a^	0.079 ± 0.002^a^	0.488 ± 0.03^a^	31.5 ± 1.13^a^
F1-III	0.596 ± 0.05^a^	5.410 ± 0.91^a^	0.532 ± 0.05^a^	0.582 ± 0.06^a^	1.668 ± 0.23^a^	0.075 ± 0.003^a^	0.479 ± 0.02^a^	32.1 ± 1.25^a^
F1-IV	0.591 ± 0.04^a^	5.088 ± 0.89^b^	0.441 ± 0.05^b^	0.579 ± 0.05^a^	1.152 ± 0.22^b^	0.062 ± 0.002^b^	0.361 ± 0.03^b^	34.9 ± 1.45^b^
F2-I	0.505 ± 0.04^a^	5.231 ± 0.95^a^	0.512 ± 0.06^a^	0.533 ± 0.05^a^	1.581 ± 0.18^a^	0.039 ± 0.002^a^	0.274 ± 0.02^a^	14.2 ± 1.12^a^
F2-II	0.511 ± 0.05^a^	5.188 ± 0.91^ac^	0.501 ± 0.06^a^	0.551 ± 0.06^a^	1.534 ± 0.21^a^	0.041 ± 0.002^a^	0.289 ± 0.03^a^	15.7 ± 1.41^a^
F2-III	0.513 ± 0.03^a^	5.022 ± 0.89^bc^	0.499 ± 0.04^a^	0.561 ± 0.05^a^	1.414 ± 0.19^a^	0.042 ± 0.003^a^	0.288 ± 0.03^a^	16.1 ± 1.10^a^
F2-IV	0.514 ± 0.03^a^	4.858 ± 0.88^b^	0.418 ± 0.05^b^	0.563 ± 0.07^a^	1.005 ± 0.24^b^	0.053 ± 0.003^b^	0.339 ± 0.03^b^	18.8 ± 1.08^b^

The values are the mean ± SD.

^a,b,c^Significantly different from each dose group for the same generation (*P* < 0.05).

The kidney weight is the weight of both kidneys.

The organ index = organ weight/body weight × 100 (mg/g).

**Table 3:** Developmental findings for mice given swainsonine by intraperitoneal injection.

	Number of live pups on PND 0 (%)	Number of live pups on PND 4 (%)	Number of live pups on PND 15 (%)	Body weights of live pups on PND 0 (g)	Body weights of live pups on PND 4 (g)	Body weights of live pups on PND 15 (g)
F0-I	80.5 ± 3.5^a^	75.21 ± 5.6^a^	73.70 ± 4.88^a^	1.60 ± 0.32^a^	2.51 ± 0.55^a^	6.87 ± 0.89^a^
F0-II	83.6 ± 4.1^a^	80.45 ± 5.1^ab^	77.34 ± 4.55^a^	1.63 ± 0.35^a^	2.67 ± 0.61^a^	6.95 ± 0.93^a^
F0-III	87.6 ± 3.6^a^	84.67 ± 5.8^b^	83.66 ± 4.18^b^	1.64 ± 0.41^a^	2.70 ± 0.71^a^	7.04 ± 0.95^a^
F0-IV	98.5 ± 5.1^b^	95.70 ± 5.9^c^	95.70 ± 5.90^c^	1.74 ± 0.44^b^	3.54 ± 0.74^b^	8.55 ± 0.88^b^
F1-I	75.5 ± 4.2^a^	70.55 ± 4.89^a^	65.66 ± 4.98^a^	1.58 ± 0.36^a^	2.31 ± 0.65^a^	6.54 ± 0.97^a^
F1-II	80.59 ± 4.07^ab^	76.48 ± 4.23^ab^	70.23 ± 4.18^a^	1.60 ± 0.42^a^	2.41 ± 0.53^a^	6.74 ± 0.92^a^
F1-III	88.33 ± 5.1^b^	85.56 ± 4.68^b^	83.56 ± 4.51^b^	1.62 ± 0.41^a^	2.58 ± 0.68^a^	6.95 ± 0.97^a^
F1-IV	98.1 ± 5.5^c^	96.70 ± 5.10^c^	96.70 ± 5.10^c^	1.75 ± 0.39^b^	3.61 ± 0.74^b^	8.31 ± 0.89^b^

The number of live pups on PND 0 (%) = number of live pups on PND 0/total number of pups born × 100 [postnatal day (PND)]. The number of live pups on PND 4 (%) = number of live pups on PND 4/total number of pups born × 100. The number of live pups on PND 15 (%) = number of live pups on PND 15/total number of pups born × 100. The sex ratio of live pups = number of live males/total number of live pups.

The values are the mean ± SD. ^a,b,c^Significantly different from each dose group for the same generation (*P* < 0.05).

**Table 4:** Reproductive findings in mice given swainsonine by intraperitoneal injection.

	Number of pairs	Number of pairs with successful copulation	Copulation index	Number of pregnant females	Fertility index	Gestation index	Gestation length (days)
F0-I	10	7.56^a^	75.6^a^	7.41	98.02^A^	83^A^	20.10 ± 1.23^A^
F0-II	10	8.34^ab^	83.4^ab^	8.11	97.24^A^	90^A^	19.85 ± 1.21^A^
F0-III	10	8.86^b^	88.6^b^	8.77	98.98^A^	96^A^	20.55 ± 1.25^A^
F0-IV	10	9.87^c^	98.7^c^	9.87	100^A^	100^A^	20.41 ± 1.36^A^
F1-I	10	7.20^a^	72.0^a^	6.32	87.78^B^	76^B^	19.87 ± 1.30^A^
F1-II	10	7.98^ab^	79.8^ab^	6.91	86.59^B^	85^B^	20.21 ± 1.29^A^
F1-III	10	8.23^b^	82.3^b^	7.32	88.94^B^	93^B^	19.98 ± 1.31^A^
F1-IV	10	9.93^c^	99.3^c^	9.93	100^A^	100^A^	20.14 ± 1.34^A^

The values are the mean ± SD. The copulation index (%) = number of copulated rats/number of pairs × 100. The fertility index (%) = number of pregnant females/number of pairs with successful copulation × 100. The gestation index (%) = number of dams with live pups/number of pregnant females × 100.

^a,b,c^Significantly different from each dose group for the same generation (*P* < 0.05).

^A,B^Significantly different from each dose group for different generations (*P* < 0.05).
